# Is IP-10 an Accurate Marker for Detecting *M. tuberculosis*-Specific Response in HIV-Infected Persons?

**DOI:** 10.1371/journal.pone.0012577

**Published:** 2010-09-07

**Authors:** Delia Goletti, Alamelu Raja, Basirudeen Syed Ahamed Kabeer, Camilla Rodrigues, Archana Sodha, Stefania Carrara, Guy Vernet, Christophe Longuet, Giuseppe Ippolito, Satheesh Thangaraj, Marc Leportier, Enrico Girardi, Philippe Henri Lagrange

**Affiliations:** 1 Translational Research Unit, Department of Epidemiology and Preclinical Research, Lazzaro Spallanzani National Institute for Infectious Diseases (INMI), Rome, Italy; 2 Department of Immunology, Tuberculosis Research Centre (ICMR), Tamil Nadu, Chennai, India; 3 Microbiology Department, P.D. Hinduja National Hospital and Research Center, Mumbai, India; 4 Scientific Direction, Fondation Mérieux, Lyon, France; 5 Scientific Direction, INMI, Rome, Italy; 6 BioMérieux, Research and Development Immunoassays, Chemin de l'Orme, Marcy L'Etoile, France; 7 Department of Epidemiology and Preclinical Research, INMI, Rome, Italy; 8 Microbiology Service, Saint Louis Hospital, Paris, France; McGill University, Canada

## Abstract

**Background:**

The suboptimal sensitivity of Interferon (IFN)-γ-based in-vitro assays, especially in immunocompromised individuals, emphasizes the need for alternative markers for diagnosing tuberculosis (TB). The objective of this study was to evaluate whether interferon-inducible protein (IP)-10, monocyte chemotactic protein (MCP)-2 and interleukin (IL)-2 can be useful biomarkers for evaluating a specific response to RD1 antigens associated to active TB disease in HIV-infected individuals.

**Methodology/Principal Findings:**

The study was carried out in India, the country with the highest TB burden in the world. Sixty-six HIV-infected individuals were prospectively enrolled, 28 with active-pulmonary-TB and 38 without. The whole blood assay based on RD1-selected peptides (experimental test) and QuantiFERON-TB Gold In tube (QFT-IT) was performed. Plasma was harvested at day-1-post-culture and soluble factors were evaluated by ELISA. The results indicate that by detecting IP-10, the sensitivity of the experimental test and QFT-antigen (75% and 85.7% respectively) for active TB was higher compared to the same assays based on IFN-γ (42.9% and 60.7% respectively) and was not influenced by the ability to respond to the mitogen. By detecting IP-10, the specificity of the experimental test and QFT-antigen (57.9% and 13.2% respectively) for active TB was lower than what was reported for the same assays using IFN-γ-detection (78.9% and 68.4% respectively). On the other side, *in vitro* IL-2 and MCP-2 responses were not significantly associated with active TB.

**Conclusions:**

HIV infection does not impair RD1-specific response detected by IP-10, while it significantly decreases IFN-γ-mediated responses. At the moment it is unclear whether higher detection is related to higher sensitivity or lower specificity of the assay. Further studies in high and low TB endemic countries are needed to elucidate this.

## Introduction

Tuberculosis (TB) is the most frequent opportunistic infection in persons with HIV infection. In 2008, there were an estimated 1.4 million new cases of TB among persons with HIV infection, and TB accounted for 26% of AIDS-related deaths [1]. Innovative diagnostic tools for TB, new and enhanced treatment strategies, and validated markers of treatment efficacy are needed to reduce the burden of TB-HIV epidemic. This needs to be shown as being useful in TB-endemic settings [2].

A recent breakthrough in the diagnosis of *Mycobacterium tuberculosis* infection has been the development of T-cell-based Interferon-γ Release Assays (IGRAs) that use antigens belonging to *Mycobacterium tuberculosis* region of difference (RD1), including early secreted antigenic target-6 (ESAT-6) and culture filtrate protein 10 (CFP-10). Two commercial IGRAs, based on overlapping peptides from CFP-10 and ESAT-6 are now available. Evidence reviewed elsewhere [3], [4] suggests that they are more specific, correlate better with *M. tuberculosis* exposure in low incidence settings and are less affected by bacillus Calmette-Guérin (BCG) vaccination than the tuberculin skin test (TST). However, although better than the TST, their accuracy in persons with HIV is still limited, particularly in “mitogen- unresponsive”[5]–[11] individuals and in those with low CD4^+^ T-cell counts.

We developed an *in vitro* IFN-γ immune diagnostic assay for active TB, the novelty of which consists of the use of multiepitopic RD1 peptides that are selected by computational analysis [12], [13]. The response to these peptides can be detected in individuals with ongoing *M. tuberculosis* replication (such as during active TB disease and/or recent infection) and decreases during TB therapy [14], [15]. Sensitivity of the assay to detect active TB is around 70% and thus needs to be improved [16]–[18]. Moreover, as for commercial IGRAs, sensitivity can be reduced in HIV-positive patients [5].

It has been recently shown that the accuracy of IGRAs may be enhanced by the addition of other *M. tuberculosis*-specific antigens [19], [20] by improving the incubation step [21]–[23] or by measuring biomarkers other than IFN-γ. In particular, it has been shown that IFN-γ-inducible protein 10 (IP-10), monocytes chemotactic protein 2 (MCP-2) and interleukin (IL)-2 may be additional biomarkers for LTBI detection after RD1-specific stimulation in both adults [24]–[28] and children [29]. IP-10 is involved in trafficking monocytes and activated Th1 cells to inflamed foci [30]. Serum and pleural fluid IP-10 levels have been evaluated as biomarkers for diagnosis, prognosis, and monitoring of treatment efficacy in inflammatory and infectious diseases including TB [31]. *M. tuberculosis* antigen-dependent IL-2 production has been demonstrated in patients with active TB [25] and its serum concentrations (that are elevated in patients with active TB) return to normal with treatment [32].

The objective of this study was to evaluate whether IP-10, MCP-2 and IL-2 can be useful biomarkers for evaluating a specific response to RD1 antigens associated to active TB disease in HIV-infected individuals. To this end, we enrolled HIV-infected patients in India, the country with the highest TB burden in the world. India accounts for one fifth of the global incidence with an estimated 1.9 million cases annually, 5% of which are HIV-co-infected [33].

## Results

### Characteristics of the enrolled individuals

We enrolled 66 HIV-infected individuals; 28 of them had active pulmonary TB (19 microbiologically confirmed and 9 clinically diagnosed). All patients with active TB were treated as a single group in the analysis. Among those without active TB, only 3/38 presented symptoms related either to oral candidiasis (2/3) or chronic liver failure with ascitis (1/3) whereas the others (35/38) were free of clinical symptoms. CD4^+^ T-cell counts and the demographic and clinical features of the enrolled participants are described in **Table 1**.

**Table 1:** Demographic and clinical characteristics of the HIV-infected individuals enrolled in the study.

	No active TB N. 38	Active TB N. 28
**Median Age (IQR)**	31.5 (27.7–40.0)	35.0 (30.5–38.0)
**Female gender (%)**	14 (36.8)	4 (14.3)
**BMI (IQR)**	19.8 (18.3–24.5)	20.0 (16.9–22.7)
**Pulmonary TB microbiological diagnosis**	–	19
**Pulmonary TB clinical diagnosis**	–	9
**TST median (IQR)**	0 (0–0)	5.0 (0–20.0)
TST negative*	30 (78.9)	13 (46.4)
TST positive*	8 (21.1)	12 (42.9)
TST unknown*	0	3 (10.7)
CD4+ T-cell/mm^3^ median (IQR)** **N. analyzed**	189 (93–455)28	139 (42–206)22
CD4+ T-cell/mm^3^ (0–100) Number of individuals (%)**	8 (21.1)	9 (32.1)
CD4+ T-cell/mm^3^ (101–200) Number of individuals (%)**	7 (18.4)	8 (28.8)
CD4+ T-cell/mm^3^ (>200) Number of individuals (%)**	13 (34.2)	5 (17.9)
**CD4+ T-cell/mm^3^ Unknown**	10 (26.3)	6 (21.4)

Footnotes: TB: tuberculosis; IQR: interquartile range; TST: tuberculin skin test; TST*: analyzed in 63/66; CD4^+^ T-cell counts**: analyzed in 50/66 individuals.

### IP-10 response to RD1 selected peptides was associated with active TB: quantitative results

**Figure 1:**
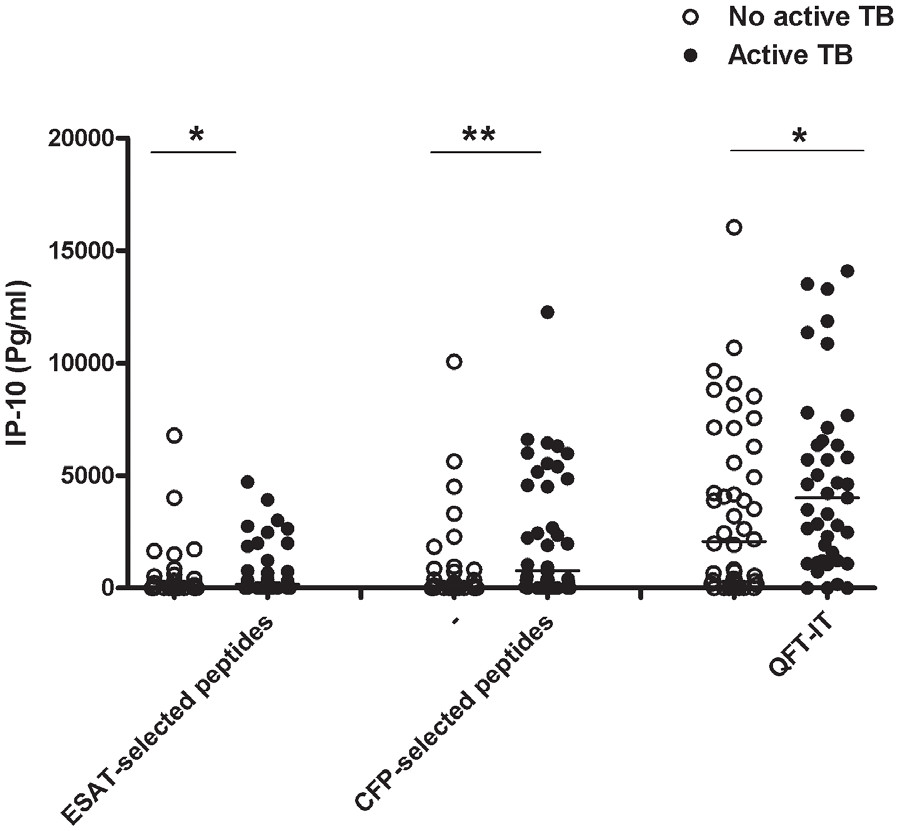
IP-10 production in response to RD1 selected peptides and QFT-antigen in HIV-infected individuals. IP-10 release in response to the RD1 selected peptides and QFT-antigen was evaluated at day 1 in the whole blood of patients with or without active TB. Horizontal lines indicate the median production. The data are presented as pg/mL. P values are reported, *: p<0.05; **:p<0.005. White circles indicate the individuals without active TB, black circles indicate the patients with active TB. IP-10 release in response to the RD1 selected peptides and to QFT-antigen was significantly higher in patients with active TB compared to those without.

The median IP-10 response to the mitogen in those with active TB (3,986 pg/ml; IQR: 2517-6097) was not significantly different from what was recorded in individuals without active TB (4,565 pg/ml; IQR: 2368-7201). On the other hand, the median response to ESAT-6 selected peptides in patients with active TB (137.4 pg/ml; IQR: 0-580.7) was significantly different than in those without active TB (6.22 pg/ml; IQR: 0-187) (p = 0.04) (**Figure 1**). Similarly, the median response to CFP-10 selected peptides in those with active TB (268 pg/ml; IQR: 0-2055) was significantly different (p<0.001) from what was recorded in those without active TB (100 pg/ml; IQR: 0.00-353). The median responses to the QFT-antigen in those with active TB (3579 pg/ml; IQR: 2270-5519) was significantly higher than in those without active TB (2682 pg/ml; IQR: 868-79-5507) (p = 0.04) (**Figure 1**). No correlation between the response to IP-10 and CD4+ T-cell counts was found (data not shown).

**Figure 2:**
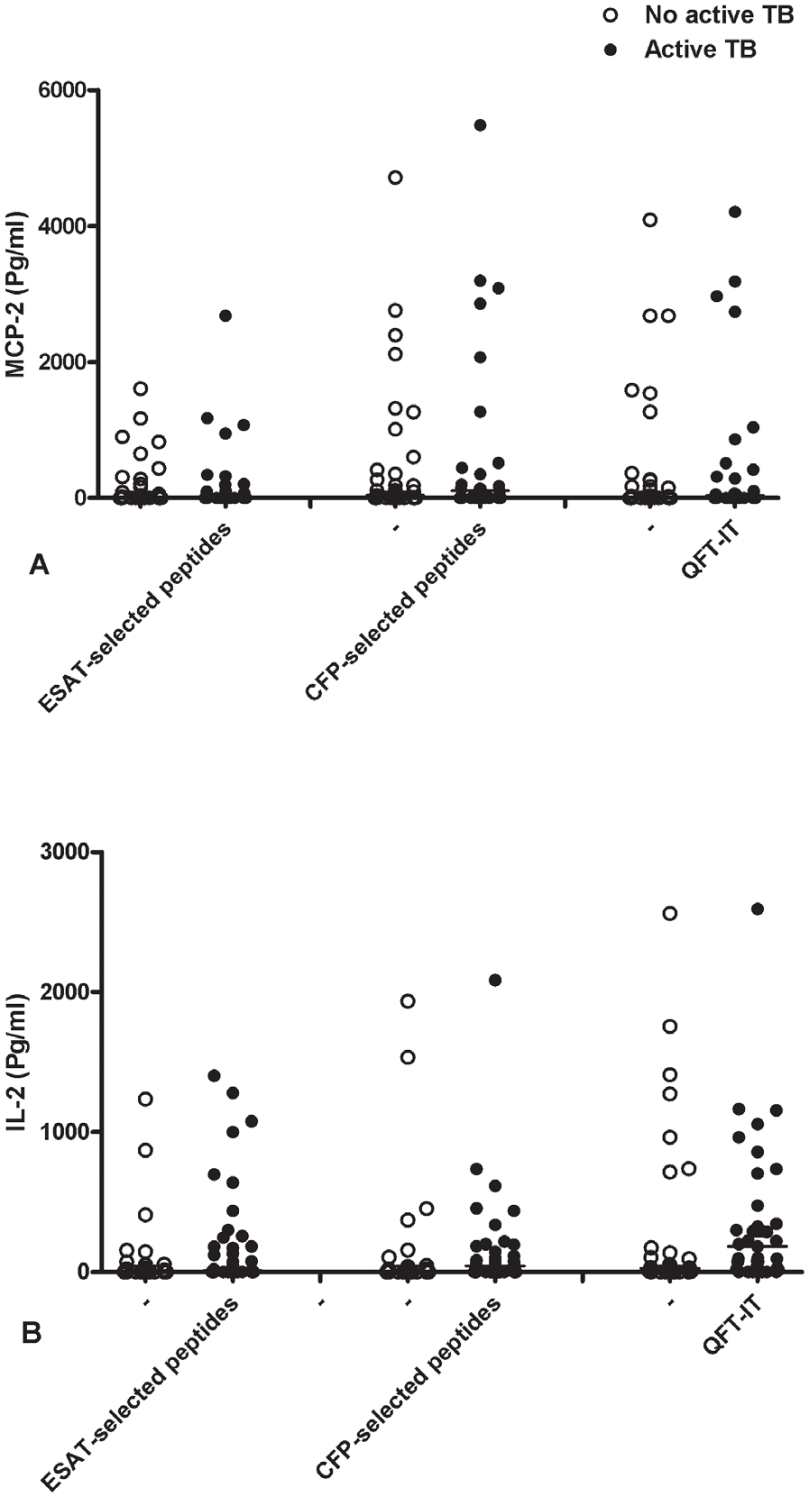
IL-2 and MCP-2 production in response to RD1 selected peptides and QFT-antigen in HIV-infected individuals. IL-2 (**A**) and MCP-2 (**B**) release in response to the RD1 selected peptides and QFT-antigen was evaluated at day 1 in the whole blood of patients with or without active TB. Horizontal lines indicate the median production. White circles indicate the individuals without active TB, black circles indicate the patients with active TB. No significant differences were found among the different comparisons performed.

No significant differences in MCP-2 and IL-2 responses were found between those with active TB and those without for all the stimuli tested (**Figure 2A–B**).

### IP-10 response to RD1 selected peptides and QFT-antigen: qualitative data

Based on the significant difference found in the quantitative analysis of the response to IP-10, we performed a receiver-operator characteristic (ROC) analysis for the IP-10 response to ESAT-6 and CFP-10 selected peptides in order to evaluate its potential use in discriminating the different stages of TB. In this analysis, we used the HIV-uninfected individuals with and without active TB as comparator groups that were enrolled in parallel, as reported in a previous study [34]. We constructed the curve using the highest response value found in response to RD1 selected peptides, either to CFP-10 or the ESAT-6 peptide pool.

**Figure 3:**
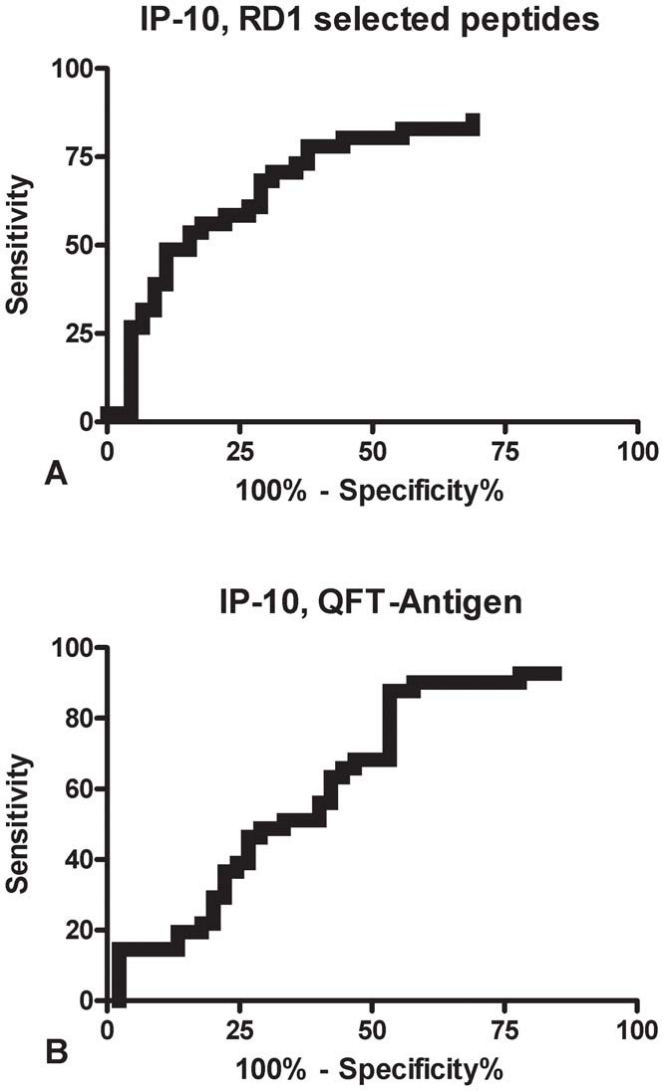
IP-10 release in response to RD1 selected peptides and TB antigen is associated with active TB. An ROC analysis was performed among the HIV-uninfected individuals using the active TB patients and the community controls as comparator groups. **A)** IP-10 release in response to the RD1 selected peptides is significantly associated with active TB. **B)** IP-10 release in response to the QFT-antigen is associated with active TB.

Significant AUC analysis results were obtained (AUC, 0.72; 95% CI, 0.61–0.83, p<0.0003) for IP-10 response to RD1 selected peptides (**Figure 3A**). For scoring purposes we chose a cut-off point to maximize the sum of sensitivity and specificity. We found that a cut-off point of 350 pg/ml predicted active TB in the HIV-uninfected individuals with 68.29% sensitivity (95% CI, 51.91%–81.91%) and 71.11% specificity (95% CI, 55.69%–83.63%).

Significant AUC analysis results were also obtained (AUC, 0.63; 95% CI, 0.51–0.75, p<0.02). for IP-10 response to the TB antigen of the QFT format (**Figure 3B**). For scoring purposes we chose a cut-off point to maximize the sum of sensitivity and specificity. We found that in HIV-uninfected individuals, a cut-off point of 698 pg/ml predicted active TB with 90.24% sensitivity (95% CI, 76.87%–97.28%) and 42.22% specificity (95% CI, 27.66%–57.85%).

By using the cut-off point identified in the ROC analysis, we found that the sensitivity and specificity of the IP-10-response to RD1 selected peptides for active TB among the HIV-infected individuals was 75.0% and 57.9% respectively (Table 2). The proportion of IP-10-positive responses to RD1 selected peptides was significantly higher in patients with active TB (21/28, 75.0%) compared to those without (16/38, 42.1) (p = 0.01).

**Table 2:** Response to the IP-10-based and IFN-γ-based assays in those with or without active TB in HIV-infected individuals.

	Active TB			No active TB		
Stimulus	Marker detected			Marker detected		
	IP-10	IFN-γ	*p* value	IP-10	IFN-γ	*P* value
	Positive over total (%)			Positive over total (%)		
**RD1-selected peptide test**						
	21/28 (75.0)	12/28 (42.9)	**0.028**	16/38 (42.1)	8/38 (21.1)	0.08
**QFT-IT**						
	24/28 (85.7)	17/28 (60.7)	0.06	33/38 (86.8)	12/38 (31.6)	**0.0001**

**Footnotes:** TB: tuberculosis; QFT-IT: QuantiFERON TB Gold In tube; IP: inducible protein; IFN: interferon; RD: region of difference.

Similarly, by using the cut-off point identified in the ROC analysis, we found that the sensitivity and specificity of the IP-10-response to the TB antigen of the QFT format for active TB among the HIV-infected individuals was 85.7% and 13.2% respectively (Table 2). The proportion of IP-10-positive responses to the TB antigen of the QFT format was not significantly higher in patients with active TB than in those without (p = 0.06) (Table 2).

### IFN-γ responses to RD1 selected peptides and QFT-antigen: qualitative data

*In vitro* IFN-γ response to the mitogen was absent in 11/28 (39.2%) of patients with active TB and in 6/38 (15.8%) of individuals without active TB, and this difference was significant (p = 0.04). Based on a cut-off point identified in the HIV-uninfected individuals [34], we found that the sensitivity and specificity of the IFN-γ assay based on RD1 selected peptides among the HIV-infected individuals were 42.9% and 78.9% respectively if evaluated on the whole population (Table 2). The sensitivity and the specificity of QFT-IT were 60.7% and 68.4% respectively.

### Comparison of IP-10 and IFN-γ responses

There was a significantly higher proportion of positive response to IP-10 (21/28) than to IFN-γ (12/28) after RD1 selected peptides stimulation (p = 0.03) among the HIV-infected individuals with active TB. However, no significant difference was found in those without active TB (16/38 vs. 8/38 respectively) (p = 0.08) (Table 2). Regarding the responses to the TB antigen of the QFT format, there was a higher proportion of positive response to IP-10 (24/28) than to IFN-γ (17/28) among those with active TB, although it was not significant (p = 0.06). Interestingly, there was a significantly higher proportion of positive response to IP-10 (33/38) than to IFN-γ (12/38) among those without active TB (p = 0.0001).

### Impact of the response to the mitogen on the score of the assays based on IFN-γ and IP-10: correlation with the CD4^+^ T-cell counts

We evaluated the impact of the ability to respond to the mitogen on the scoring of IFN-γ- or IP-10-based response to either the RD1 selected peptides or TB antigen of the QFT format. Among the 11 subjects with active TB classified as “mitogen-unresponsive”, only 18.2% (2/11) responded to the assays based on IFN-γ in response to RD1 selected peptides compared to 58.8% (10/17) of the “mitogen-responsive”, and this difference was significant (p = 0.02). Similar results were found for QFT-IT, with 36.4% (4/11) of those classified as “mitogen-unresponsive” vs. 76.5% (13/17) of those defined as “mitogen-responsive” and this difference was close to significance (p = 0.052). Differently, regarding the responses to IP-10 among those classified as “mitogen-responsive”, 76.5% (13/17) responded to RD1 selected peptides, similar to the 72.7% (8/11) defined as “mitogen-unresponsive” (p = 1). Similar results were found for the response to the TB-Antigen of the QFT-IT format, 76.5% (13/17) of those defined as “mitogen-responsive” vs.100% (11/11) of those defined as “mitogen-unresponsive” (p = 0.13).

Among the patients without active TB, 15.6% (5/32) of the subjects classified as “mitogen-responsive” responded to the assays based on IFN-γ in response to RD1 selected peptides compared to 50.0% (3/6) of the “mitogen-unresponsive” (p = 0.09). Similar data were obtained in response to QFT-IT, 28.1% (9/32) vs. 50% (3/6) (p = 0.35), respectively. Regarding the responses to IP-10 among those defined as “mitogen-responsive”, 37.5% (12/32) responded to RD1 selected peptides vs. 66.7% (4/6) of those defined as “mitogen-unresponsive”. Similar results were found for the response to the TB-Antigen of the QFT-IT format, 84.4% (27/32) vs. 100% (6/6) (p = 0.57).

Finally, we evaluated whether there was a difference between the CD4^+^T-cell counts of those defined as “mitogen- responsive” and “mitogen-unresponsive”. We found that among all the patients tested, the CD4^+^T-cell counts were significantly lower in those defined as “mitogen-unresponsive” (median: 41; IQR: 23-184) than in the “mitogen-responsive” subjects (median: 177; IQR: 95-366) (p = 0.03).

### Evaluation of test response in relation to the CD4^+^ T-cell count

**Figure 4:**
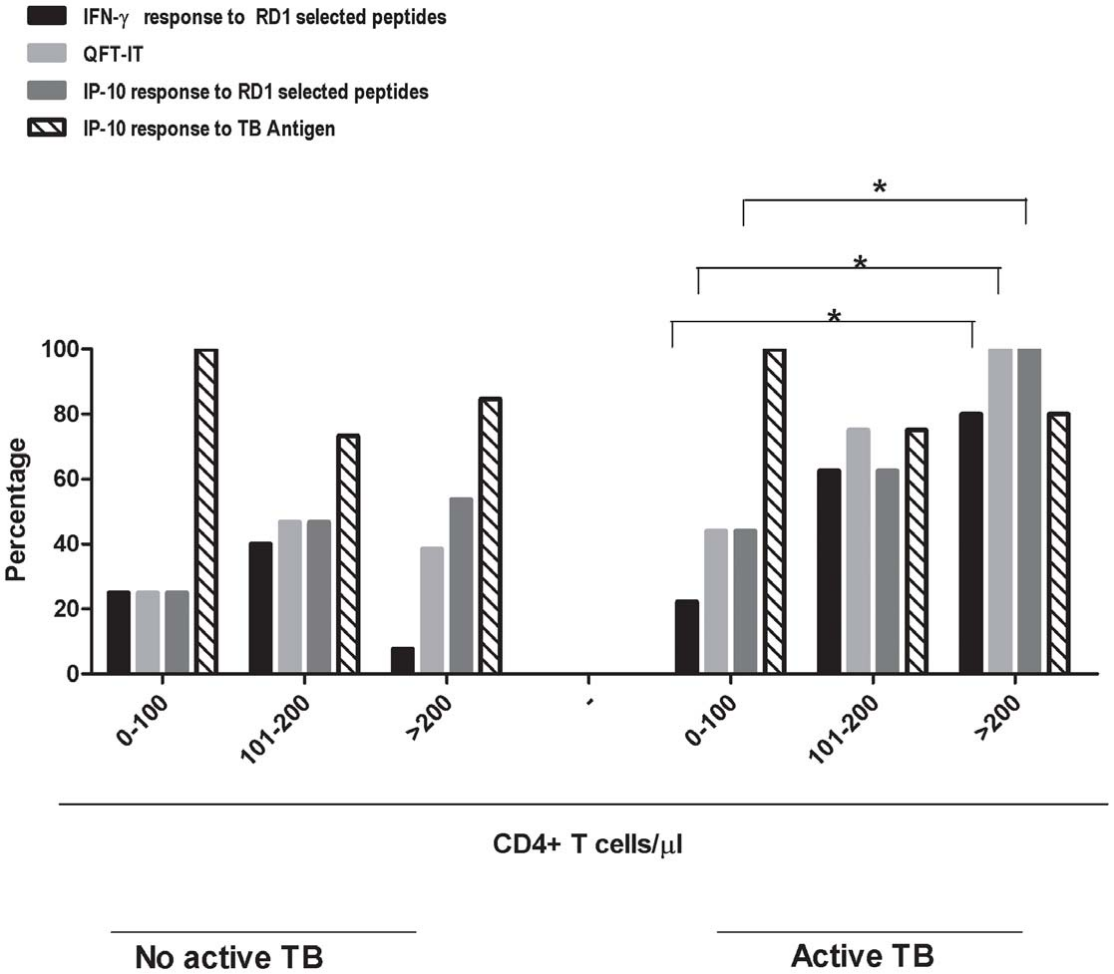
Percentage of positive response to IFN-γ and IP-10-based tests in response to RD1 selected peptides and QFT-antigen in HIV-infected individuals stratified in 3 CD4^+^ T-cells categories: i) between 0–100/µl, ii) 101–200/µl, and iii) above 200/µl. P values are reported, *: p<0.05. Among those with active TB, there was a significant difference by χ^2^ for the trend between the categories of the CD4^+^ T-cell counts and the proportion of responders to the IFN-γ-based experimental test (p = 0.032), IP-10-based experimental test (p = 0.048) and for the IFN-γ-response to the TB antigen of the QFT-IT.

To evaluate the impact of the CD4^+^ T-cell count on the response to the TB tests in those with HIV infection in detail, we stratified the CD4^+^ T-cell count into 3 categories: i) between 0–100/µl, ii) 101–200/µl, and iii) above 200/µl (**Table 1**). Among those with active TB, there was a significant difference by χ^2^ for the trend among the categories of the CD4^+^ T-cell counts in the proportion of responders to the IFN-γ based experimental test (p = 0.03), IP-10-based experimental test (p = 0.048) and for the IFN-γ-response to the TB antigen of the QFT-IT. Conversely, no significant difference was found among those without active TB (data not shown) in any of the comparisons performed (**Figure 4**).

## Discussion

We are presenting the results of a prospective study conducted in HIV-infected individuals in India (where TB is highly endemic) that was designed to investigate if factors other than IFN-γ, such as IP-10, MCP-2 and IL-2 may improve detection of the response to RD1 antigens for the immunodiagnosis of active TB.

The results of this study demonstrate that besides IFN-γ, the IP-10 response to RD1 selected peptides is associated with active TB in HIV-infected subjects. There was a significantly higher proportion of responders to IP-10 than to IFN-γ. Response to RD1 selected peptides detected by either IP-10 or IFN-γ was dependent on the CD4^+^ T-cell counts and IFN-γ mitogen response.

Regarding the response to the TB Antigen of the QFT-IT format, we found that the sensitivity of IP-10 for detecting active TB was similar to what was reported for the QFT-IT response (evaluated by definition by IFN-γ).However, differently from QFT-IT, the IP-10-mediated response was not dependent on the CD4^+^ T-cell counts and IFN-γ mitogen response. Moreover, there was a significantly higher proportion of responders to IP-10 than to QFT-IT in those without active TB, leading to a low specificity for detecting active disease.

Finally, MCP-2 and IL-2 release was not significantly associated with active TB in response either to the RD1 selected peptides or to the TB Antigen of the QFT-IT format.

In this study, we confirmed the data generated in Italy and Africa; that the *in vitro* IFN-γ response to RD1 selected peptides is associated with active HIV-TB and it is more specific than QFT-IT and/or the response to RD1 intact proteins [5], [15]. However, here we showed that the accuracy (in terms of sensitivity for active TB detection) of this test is poor, especially in those defined as “mitogen-unresponsive” and in those with low CD4^+^ T-cell counts. In respect to the previous studies, this diversity may be related to the use of the antiretroviral therapy in the Italian cohort (36% were receiving antiretrovirals) [5] that may have generated a higher immunological ability to respond, and/or to the use of the ELISPOT as a read-out test, which is characterized by a greater response detection performance than the ELISA [12].

The proportion of responders to QFT-IT in HIV-infected individuals is similar to what has already been reported in the literature [6]–[8], [11] and was significantly impaired in those defined as “mitogen-unresponsive” compared to the “mitogen-responsive”, as previously shown [reviewed in 11].

IP-10 detection improves the sensitivity for active TB in the IFN-γ-based tests in response to the RD1 selected peptides and to QFT-IT, independent of the ability to respond to the mitogen, as previously shown by others [35]. This higher sensitivity may be due to the fact that IP-10 is mainly secreted by monocytes/macrophages [30] while IFN-γ and IL-2 are secreted mainly by CD4^+^ T-cells. Hence, IP-10 has the probability of being less affected by HIV infection and less influenced by low CD4^+^ T-cell counts. However IP-10-based tests, especially those based on QFT Antigen, show a lower specificity for detecting active TB. This result may be due to the fact that IP-10 is detected in contacts of patients with active TB (in both adults [28], [34] and children [29],) and its levels in healthy contacts are not significantly different than in those with active TB [34]. These results may be important to underline the high sensitivity of IP-10 in detecting *M. tuberculosis* infection compared to IFN-γ. In fact, although active TB was excluded from the HIV-infected individuals of the study population classified as being without active disease, it was impossible to rule out contact with *M. tuberculosis* in a country like India.

Therefore it is unclear whether the higher proportion of positive IP-10 results is related to a higher sensitivity in detecting “infection per se” or to a lower specificity of the assay.

The specificity of the assay based on RD1 selected peptides is higher when evaluated either by IFN-γ or IP-10 detection, compared to QFT-IT, and this is not unexpected. In fact, QFT-IT uses a greater variety of epitopes to elicit *M. tuberculosis* immune responses by effector memory T-cells and is based on pools of overlapping peptides spanning the whole length of CFP-10 and ESAT-6 proteins and an additional peptide from RD11 [3], [4]. Conversely, the selective approach of the design of the test based on RD1 selected peptides reduces the false positive test results among those without active TB, with a loss of diagnostic sensitivity for the detection of active TB [12]–[18]. Whether it is more acceptable to have false positive test results that may lead to over treatment, or false negative test results that could potentially lead to missing cases with active TB to be treated is a matter of debate and is largely dependent upon the prevalence of *M. tuberculosis* infection and the pre-test probability of TB in a community.

According to the literature generated in HIV-uninfected children [29], in this study on HIV-infected adults we observed that the stimulation index of IP-10 was lower than that of IFN-γ, despite the higher levels of cytokines produced. This was due to the higher background level of IP-10 in unstimulated samples compared to IFN-γ (data not shown).

The rate of indeterminate results in our study is 25.7% (17/66), similar to the rates observed in other studies in which patients with active TB disease [5]–[8], [ and reviewed by 11] and those not receiving antiretroviral treatment have similar CD4^+^ T-cell counts. It is worthwhile to note that IFN-γ response to either RD1 selected peptides or QFT-IT was detected in a small proportion of patients defined as mitogen-unresponsive. This may depend on fact that a better cut-off point for the mitogen should be provided in HIV-uninfected subjects as suggested by Harada [36] and in HIV-infected persons, as suggested by Syed Ahamed Kabeer [35]. The new CDC guidelines for using IGRAs indicate that besides the mitogen score, the QFT-IT is scored positive based on the specific response to the antigen [37]. In addition, the IP-10-based assays are scored positive in the majority of the individuals classified as “mitogen-unresponsive”.

It is also important to take into account that we found that the cut-off point for IP-10 in response to the QFT-antigen in this study performed in India is very similar (698 pg/ml) to what was reported by Ruhwald (673 pg/ml) in studies conducted in Europe with another detection readout [27]. Moreover, the sensitivity of the assay for active TB diagnosis in the HIV-uninfected individuals in our study was 90%, close to what was reported by Ruwald [27]. These data are very interesting and may indicate the solidness of the IP-10-based assay worldwide. Differently, the response of MCP-2 to the QFT-antigen did not show any diagnostic value. This data is different than what was previously shown in a European setting [27] and at the moment, the reason is unclear.

Kabeer at al [35] obtained similar results among the HIV-infected individuals, however in that study, the cut-off point was found in a less stringent way than in this study and only few data regarding the CD4^+^ T-cell counts were available.

The study has some limitations. Subjects were selected according to a certain TB status and comparison between the *in vitro* assays and the CD4^+^ T-cell counts were made, although CD4^+^ T-cell counts were not performed on the total population. Nonetheless, although BCG coverage in India is high and we may expect that the majority of the population studied is BCG-vaccinated [38], the BCG status of a large number of the individuals (96.4%) was unknown. However, despite these limitations, the prospective design of the study, the evaluation of 6 *in vitro* assays for TB diagnosis (5 experimental and 1 commercial) and the relatively large number of HIV-infected individuals enrolled to evaluate the performance of the different immune based tests in clinical practice render the results solid.

In conclusion, we show that HIV infection significantly impairs the IFN-γ response to the mitogen and RD1 tests in those with active TB, with little impact on IP-10 responses. At the moment it is unclear whether higher detection of IP-10 is related to higher sensitivity or to lower specificity of the assay. Further studies are needed to elucidate it in both high endemic and low endemic TB countries.

## Materials and Methods

### Population of patients

Study individuals were prospectively recruited from April, 2007 to March, 2008 in India at: i) Tuberculosis Research Centre, Chetput, Chennai and ii) P.D.Hinduja National Hospital and Research Center, Mumbai. The study was approved by the local ethical committees: the Institutional Ethical Committee of Tuberculosis Research Centre in Chennai (TRC-IEC No: 2006005) and the Institutional Review Board at Hinduja Hospital, Mumbai (No: 316 -05-CR). Informed signed consent was required to participate to the study. Medical information and heparinised blood were obtained from individuals at enrolment after signing an informed consent. For this particular study however, we did not use the results from the Mumbai site because the data were incomplete in terms of CD4^+^ T-cell counts and detection of biomarkers different from IFN-γ.

The demographic details and information on previous tuberculin skin test (TST) results were collected. Individuals with a previous history of TB, silicosis, end stage renal disease, leukaemia/lymphoma, who had TST in the past 16 months or who had received anti-TB therapy or immunosuppressive therapy for more than two weeks were excluded from the study. Pregnant and lactating patients were also excluded.

Active TB was defined as microbiologically confirmed if a sputum smear was positive for acid-fast bacilli (AFB) on microscopy by Ziehl-Neelsen method and/or *M. tuberculosis* was identified in sputum culture in conventional Lowenstein Jensen (BioMérieux Inc., Marcy I'Etoile, France) and/or in liquid BacT/ALERT MP medium (BioMérieux Inc., Marcy I'Etoile, France). Conversely, patients were classified as having “clinical TB” if the diagnosis was based on clinical and radiologic criteria (after excluding other diseases) including appropriate response to anti-tuberculosis therapy.

Subjects classified as “no TB” were free of TB symptoms and were checked to have a normal chest X-Ray and to be AFB smear and culture negative. These individuals did not report having had close contacts with patients with active TB in the past and at this time of the study. The presence of HIV infection was evaluated by two ELISA (Retroquic Comb Aids-RS, Span Diagnostics, India and HIV TRI-DOT, J. Mitra & Co, India) in serum.

### Tuberculin Skin Test

TST was performed by intradermally injecting 2 TU (tuberculin unit) of purified protein derivative (PPD) RT23 (Staten Serum Institut, Copenhagen, Denmark) by Mantoux method; the induration was measured by trained professionals between 48–72 hrs after PPD injection. The cut-off point for TST positivity was considered as 5 mm for the HIV-infected individuals in accordance with Indian guidelines [39].

### RD1 selected peptides and stimuli used for cell cultures

The selection of Human Leukocytes Antigens (HLA)-class II restricted epitopes of ESAT-6 and CFP-10 *M. tuberculosis* proteins was performed by a quantitative implemented HLA peptide-binding motif analysis as previously described for ESAT-6 [12], [13]. The whole blood test was carried out as previously described [34]. The two centres were provided with RD1 selected peptides from the same batch, detailed protocol and both received personal training from INMI's laboratory staff. Inter-site communication was present throughout the study to solve any potential problems. Clinicians were blinded to the laboratory test results and the laboratory staff was blinded to the status of the patients. Throughout the test, the assay based on RD1 selected peptides is also called “experimental test”.

### IP-10, MCP-2, IL-2 and IFN-γ ELISA

The levels of IP-10, MCP-2 and IL-2 were measured in the plasma of whole blood stimulated with or without the mitogen, RD1 selected peptides and QFT-Antigen, using Duoset ELISA Development kits as per the manufacturer's instructions (R&D Systems Inc, MN, USA). To detect the chemokines, plasma was diluted 1∶10 as a starting dilution. Further dilutions were performed when necessary. The levels of IFN-γ were evaluated by a commercial ELISA (CMI, Cellestis Limited, Carnegie, Victoria, Australia). In general, the data from stimulated whole blood reported in the text and figures are reported after the subtraction of the relative unstimulated control, which is either the whole blood with the same concentration of Dimethyl Sulfoxide (DMSO) used to dissolve the peptides for the RD1 stimulated conditions [12], [13], [34] or the “nil” for the QFT antigen.

### Commercially available assays

QFT-IT (Cellestis) was performed and the results were scored as indicated by the manufacturers. In particular, the results were scored as indeterminate if the IFN-γ response to the mitogen after subtracting the nil IFN-γ response was <0.5 IU/ml or if the nil IFN-γ response was >8 IU/ml.

### Statistical analysis

The median and range of values were calculated. The Mann-Whitney U test was used to compare continuous variables, and Chi square was used for categorical variables. Analysis was carried out with SPSS v 14 for Windows (SPSS Italia SRL, Bologna, Italy). Receiver-operator characteristic analysis was performed using Prism 4 software (GraphPad PRISM, version 4.03, La Jolla, CA, USA).
